# The Impact of the Composition on the Properties of Simulated Lunar Mare Basalt Fibers

**DOI:** 10.3390/ma17092043

**Published:** 2024-04-26

**Authors:** Jin Liu, Lida Luo, Jiali Xu, Xiaoxu Zhu, Guoying Shi, Qingwei Wang

**Affiliations:** 1 State Key Laboratory for Modification of Chemical Fibers and Polymer Materials, College of Materials Science and Engineering, Donghua University, Shanghai 201620, China; lj@dhu.edu.cn (J.L.); xujiali@dhu.edu.cn (J.X.); 2 Engineering Research Center of Advanced Glass Manufacturing Technology, Ministry of Education, Donghua University, Shanghai 201620, China; wqwq888@dhu.edu.cn; 3 College of Chemistry and Chemical Engineering, Donghua University, Shanghai 201620, China; zhuxx@dhu.edu.cn

**Keywords:** simulated lunar mare basalt fibers, glass network structure, Raman spectroscopy, area normalization

## Abstract

Lunar mare basalt is recognized as an important in situ resource on the lunar surface. However, the significant compositional variability of lunar mare basalts introduces uncertainties concerning the potential for their use in fabricating fibers and composite materials. This study investigates the impact of different components on the fiber-forming capabilities of mare basalts by simulating the compositions of basalts collected from several well-known lunar missions and then preparing simulated lunar mare basalt fibers. Raman spectroscopy is primarily employed for analysis and characterization, using “peak area normalization” to explore the impact of compositional fluctuations in the simulated lunar mare basalts on the glass network structure. The findings indicate that an increase in the Fe content raises the likelihood of basalt fibers crystallizing. Additionally, Fe^3+^ is shown to substitute for Si and Al in constructing bridging oxygen bonds in the network structure, albeit reducing the overall polymerization of the network. Meanwhile, Fe^2+^ acts as a network modifier to enhance the mechanical properties of the fibers.

## 1. Introduction

The Moon is the first extraterrestrial body where humans have successfully landed and conducted on-site exploration. A lunar base is critical for providing a testbed for the development of science and technology in areas such as telecommunications, human life support, energy systems, and research on radiation and microgravity [1]. The lunar surface is composed of lunar highlands and maria, with the lunar maria offering relatively flat and ideal locations for establishing lunar bases [2,3]. As science and technology continue to advance, utilizing the abundant in situ resources of the lunar maria, such as basalt and solar energy [4,5], can provide continuous and cost-effective raw materials and energy for lunar base construction materials like basalt glass and basalt fibers [6,7].

Basalt-derived products stand out for their remarkable cost-effectiveness, with basalt fibers leading the way due to their superior mechanical stability [8,9], resilience to extreme temperatures, and corrosion resistance. This versatility has paved the way for their widespread application across diverse sectors, including construction reinforcement, geogrids, textiles, and various insulation and composite materials [10,11,12,13]. Lunar basalt, mirroring its terrestrial counterpart, boasts not only a plentiful reserve but also the feasibility for direct utilization on the lunar surface, thereby offering a valuable resource for the construction of future lunar habitats [14].

Since the historic Apollo 11 mission in 1969, which marked the first human landing on the lunar maria, lunar exploration has brought back significant amounts of lunar soil samples, enriching our understanding of the Moon’s geology [15,16]. Notably, the Chang’e-5 mission in 2020 returned with 1731 g of predominantly basaltic lunar soil, underscoring basalt’s prominence on the lunar surface [17,18,19]. Lunar basalt’s unique oxide composition, characterized by lower alkali metal oxides and variable iron oxide content, differentiates it from its earthly counterparts [20,21,22]. This variability in composition has profound implications for the physical and chemical properties of basalt fibers and their derived materials, making the study of these effects on their structural and mechanical properties crucial for harnessing extraterrestrial resources for human use [23,24].

This research synthesized simulated lunar mare basalt fibers using samples from renowned missions, including the American Apollo series (A14, A16, A17), the Soviet Luna series (L20, L24), and China’s Chang’e-5 (CE5), to perform a comprehensive component analysis. We employed an optimized Raman spectroscopy peak-fitting method tailored to basalt fibers. By integrating differential scanning calorimetry, spectrophotometry, and nanoindentation techniques, our study elucidates the impact of compositional variations on the formation of the glass network and the mechanical attributes of these simulated lunar mare basalt fibers. The findings serve as a foundational reference for the prospective in situ exploitation of lunar mare basalt resources.

## 2. Materials and Methods

### 2.1. Preparation of Basalt Fibers

**Figure 1:**
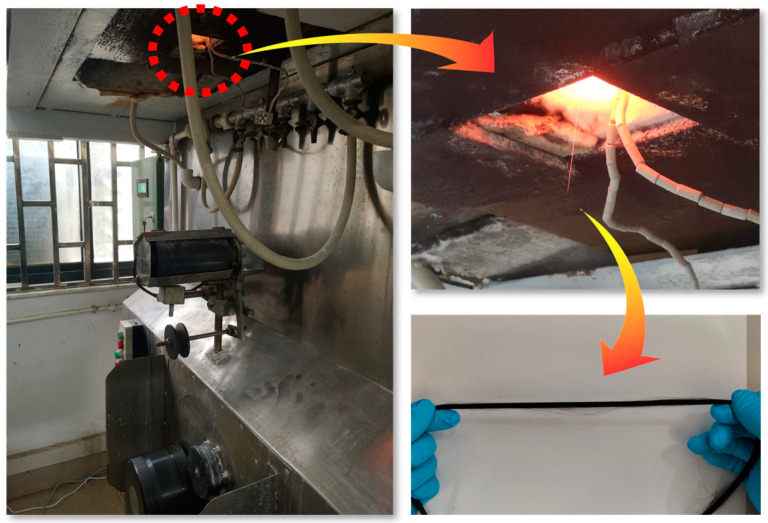
Basalt fiber drawing device.

Based on the basalt information collected from various lunar missions as cited in references [25,26,27,28,29,30,31], and to enhance the experimental study of the effects of several specific oxides on the glass network structure, this work has eliminated the uncertainties introduced by trace elements. The compositions of the simulated mare basalt samples prepared are shown in Table 1, with all the materials being of analytical-grade purity (Sinopharm, Beijing, China). The raw materials are mixed evenly and heated to 1500 °C in a high-temperature atmosphere furnace for 2 h. Subsequently, the mixture is poured into water to obtain quenched material, dried, and finally, as shown in the Figure 1, melted and drawn into basalt fibers in a platinum crucible single-filament furnace at atmospheric pressure and 1350 °C.

### 2.2. Methods

After grinding the basalt samples into 200-mesh powder, the crystallinity of the samples was tested using an X-ray diffractometer (XRD, D8 ADVANCE, Bruker, Billerica, MA, USA), with a scanning range of 10 to 80°, a step size of 0.03°, and an exposure time of 0.1 s. The thermal properties of the glass samples were analyzed using a differential scanning calorimeter (DSC, TGA/DSC 3+, Mettler Toledo, Greifensee, Switzerland), heating from room temperature to 1200 °C at a rate of 10 °C/min under a nitrogen atmosphere. The glass network structure of the samples was examined using a confocal laser Raman spectrometer (inVia Reflex, Renishaw, Wotton-under-Edge, UK), with the parameters including a 532 nm green-light source, a scanning depth of 2 μm beneath the focused surface, a scanning range of 100 to 1500 cm^−1^, an exposure time of 30 s, and a laser intensity of 50%, repeated three times. According to the ISO 14719-2011 standard [32], the absorption spectrum of the orange–red complex formed by Fe^2+^ and ortho-phenanthroline was measured using a UV–visible spectrophotometer (UV–Vis, 722N, Shanghai Precision Instrument, Shanghai, China), with a 510 nm light source, for further calculation of the iron ion valence state. The hardness, modulus, and surface stress layer thickness of the fibers were tested using a nanoindenter (Nano Indenter, G200X, KLA, San Diego, CA, USA) equipped with a cube-corner indenter, under the parameters of a maximum load force of 5 mN and a maximum depth of 5000 nm. Additionally, the tensile strength of individual fiber strands was measured using a single-fiber tensile tester (YG005A, Baien, Wenzhou, China), with a gauge length of 20 mm and a stretching rate of 5 mm/min, with each sample measured at least 30 times for valid data.

## 3. Results and Discussion

### 3.1. Crystalline Properties

**Figure 2:**
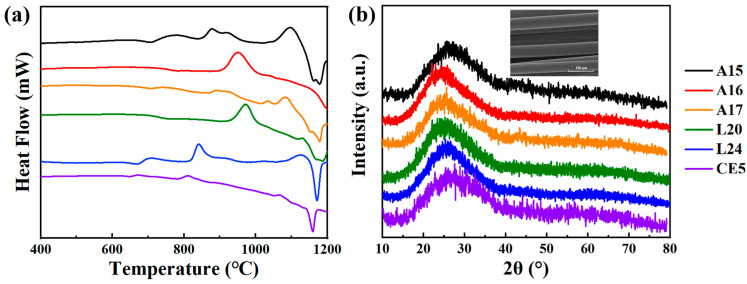
DSC and XRD patterns of the simulated lunar sea basalt: (**a**) DSC image; and (**b**) XRD image.

The DSC test was initially performed on the simulated lunar basalt fiber samples. Figure 2a displays the DSC curves for various simulated lunar basalt samples, each showing distinct glass transition temperatures (T_g_) and crystallization peak temperatures (T_c_). The difference (ΔT) between T_g_ and T_c_ serves as an indicator of the basalt fibers’ resistance to crystallization, with smaller ΔT and T_c_ values indicating a higher likelihood of crystallization in the basalt [13]. Moreover, the spectra reveal the presence of multiple T_g_s and T_c_s in most samples, introducing additional uncertainty concerning basalt crystallization. As presented in Table 2, samples CE5 and L24 demonstrate smaller ΔT and T_c_ values, whereas A16 and L20 exhibit relatively higher ΔT and T_c_ values, accompanied by smoother curves (indicating a clear T_g_ and T_c_ at only one point). This phenomenon is related to the iron content of the samples. Higher iron contents have been proven to cause liquid–liquid phase separation in the glass melt, which further exacerbates the tendency for spontaneous crystallization during the glass formation process [33], thus complicating the production of basalt fibers. Nevertheless, all the fiber samples derived from the selected simulated lunar basalt formula for this study were successfully manufactured, as evidenced by the XRD spectra in Figure 2b, which exhibit characteristic amorphous peaks. This confirms that the molten basalt samples were in a glass state, thereby minimizing potential errors from crystallinity in the subsequent Raman spectroscopy and mechanical property assessments [34].

### 3.2. Glass Network Structure

**Figure 3:**
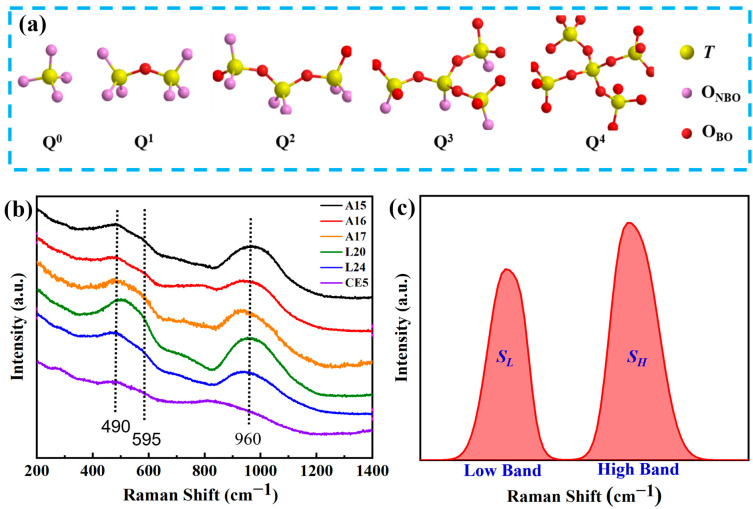
Raman spectral curve and peak division diagram of a basalt sample: (**a**) schematic diagram of Q*^n^*; (**b**) Raman curve of basalt fiber; and (**c**) peak-splitting diagram.

Raman spectroscopy is esteemed for its precise, non-invasive capability to elucidate the structural and chemical modifications within solids [35]. Within the scope of silicate glass systems, Raman spectral analysis conventionally divides the spectrum into low, mid, and high wavenumber regions [36]. The peaks within the low wavenumber domain are predominantly related to the bending vibrations of T–O–T bridging oxygen bonds (O_BO_) [37], where T″ symbolizes network-forming elements such as silicon (Si), aluminum (Al), and iron (Fe). The high wavenumber peaks, on the other hand, provide a richer depiction of the glass structure, largely attributed to the stretching vibrations of T–O–M non-bridging oxygen bonds (O_NBO_) associated with [TO4] tetrahedral units (Q*^n^*). This portion of the spectrum is crucial for discerning changes in the tetrahedral bond angles, T–O bond lengths, and force constants, which are indicative of the bond energies [38,39,40]. In this context, M″ denotes network-coordinating modifier ions like sodium (Na), calcium (Ca), and magnesium (Mg), and *n*″ represents the count of bridging oxygens. Distinct Q*^n^* species, such as Q^0^, Q^1^, Q^2^, Q^3^, and Q^4^, are delineated, as evidenced in Figure 3a. The mid-wavenumber spectrum typically presents a more subdued profile, with the few Raman peaks observed primarily extending from low wavenumber T–O_BO_ transitions or marking the onset of high wavenumber T–O_NBO_, albeit not discounting the characteristic peaks of certain chemical bonds [41].

Raman spectroscopic analysis was performed on the synthesized simulated lunar basalt fiber samples, as depicted in Figure 3b, which uniformly showcased the typical Raman peaks characteristic of silicate glass. The spectral data distinctly delineated the low-frequency (330–630 cm^−1^) and high-frequency (800–1200 cm^−1^) bands. Given that oxygen atoms in amorphous structures are exclusively present as either bridging or non-bridging oxygen bonds, and as their cumulative proportion amounts to 100%, we use the Origin software (2021 version) to perform linear fitting on the Raman spectra. By employing the “peak area normalization” method, the sum of the peak areas in the low- and high-wavelength regions is made equal to one. The determination of the proportions of the low-frequency peak area (*S_L_*) and high-frequency peak area (*S_H_*) was facilitated through Gaussian peak deconvolution, as illustrated in Figure 3c. Typically, an increase in the T–O–T bridging oxygen bonds within the basalt fiber network correlates with a higher polymerization degree. Table 3 outlines the ratio of fitted peak areas in the low-frequency to high-frequency bands for each analyzed basalt specimen. It is imperative to note that silicate samples from identical systems undergo consistent baseline correction and peak deconvolution methodologies. The peak positions for each constituent are expected to fall within the analogous Raman shift ranges, ensuring the deconvoluted curve precisely aligns with the empirical Raman spectral curve’s amplitude.

As elucidated earlier, the low-frequency Raman peaks primarily originate from the vibrations of bridging oxygen bonds (O_BO_). In this study, we observed that all the fabricated simulated lunar basalt fiber samples presented peaks at approximately 490 cm^−1^ within this spectrum, aligning with the symmetric bending vibrations of the Si–O–Si bridging oxygen bonds. Additionally, the notable feature around 595 cm^−1^ is ascribed to the asymmetric bending vibrations of O_BO_ in the tetrahedral structures, indicating the presence of differing elements at either terminus of the O_BO_ [42]. The observed variations in the peak intensities and breadths at these spectral positions imply that the alterations in the T–O–T bond angles are modulated by the shifts in the chemical composition [43]. For instance, the spectral analysis of sample CE5 revealed that the peak intensities at 490 cm^−1^ and 595 cm^−1^ are comparably similar, suggesting that apart from silicon, other network-forming elements are present in significant concentrations, supplanting one of the silicon atoms in the Si–O–Si structure and thereby inducing the asymmetric bending vibrations of O_BO_.

**Figure 4:**
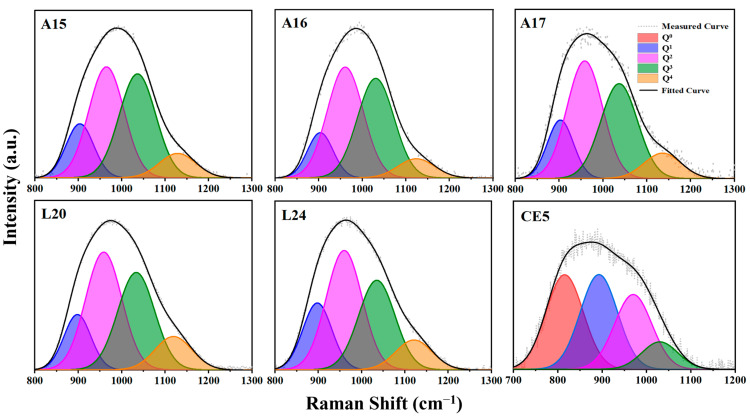
Fitting of each Q*^n^* peak of a simulated lunar sea basalt fiber.

The high-frequency Raman peaks, indicative of O_NBO_ vibrations, are analyzed through Gaussian deconvolution, anchored in the classification of the Q*^n^* tetrahedral units. The prevalence of Q*^n^* configurations within the glass matrix is contingent upon the sample’s chemical composition. The Raman peaks’ shift, area, and breadth serve as proxies for the quantity of the non-bridging oxygen bonds and the glass’s polymerization degree. Adhering to methodologies delineated in references [44,45], the high-frequency spectral data for each specimen were subjected to deconvolution using five Gaussian peaks corresponding to Q*^n^* units (where *n* = 0 to 4). The optimization of the peak positions for each Q*^n^* component was iteratively refined to produce the results illustrated in Figure 4. From the deconvolution of the high-frequency peaks, it is inferred that an increase in the Q^3^ and Q^4^ content correlates with an elevated network polymerization degree, whereas an abundance of Q^0^ and Q^1^ signifies a reduced polymerization level [46,47].

Comprehensive analysis of the aggregated Raman spectral components within the samples underscores the significance of iron (Fe) as a characteristic element of basalt. Predominantly, Fe’s behavior, contingent upon its oxidation state, oscillates between network-forming and network-modifying roles [48]. In its trivalent form, iron oxide (Fe^3+^) acts as a network intermediate, adopting various coordination modes within network-forming entities [FeO*_x_*] (*x* = 4, 5, 6), and simultaneously functions as a modifying agent. Conversely, divalent iron (Fe^2+^) typifies a network modifier [49,50,51]. Consequently, Fe’s valence state is pivotal to defining the structural network of basalt fibers. Throughout the high-temperature melting phase of basalt fiber production, a fraction of Fe_2_O_3_ undergoes reduction. The iron reduction index (Fe^2+^/ΣFe, IRI) for each sample was ascertained and computed utilizing spectrophotometric techniques. Table 4 enumerates the IRI values for the simulated lunar basalt fibers alongside the quantification of iron oxides across varied oxidation states.

In the preceding context, it was mentioned that CE5 exhibits peaks at 490 cm^−1^ and in proximity to 595 cm^−1^ in the low-frequency range, with their intensities being quite close. This may be attributed to the asymmetric bending vibration of Fe–O–Si(Al) bridging oxygen bonds. Although there is considerable debate regarding the analysis of the iron content’s impact on the glass structure solely through Raman spectroscopy, researchers still endeavor to extract information regarding the iron content and its valence states from Raman spectra. Muro et al. [51] investigated the sensitivity of Fe in alumino-silicate glass systems using Raman spectroscopy and found that when the aluminum content is high, the detection of iron–oxygen bonds becomes challenging. However, with a lower aluminum content and an increasing iron content, the competition of Fe^3+^ with O_BO_ gradually becomes detectable in Raman spectra. The characteristic spectra of [FeO_4_] tetrahedra in Raman spectra are mainly reflected in the Q^1^~Q^2^ unit segments. Additionally, some researchers [52,53,54] have proposed that an increase in the Fe^2+^ content gradually enhances the peak near 800 cm^−1^, while an increase in Fe^3+^ also causes a noticeable shift in the Raman peak toward lower wavenumbers in the high band. From Figure 2b, it can be observed that the CE5 sample with a higher ferrous iron content (5.9 mol%) exhibits a prominent peak near 815 cm^−1^. Simultaneously, when combined with the substantial enhancement represented as Q^0^ in Figure 3, this is because Fe^2+^ only acts as a network modifier, forming O_NBO_ after binding with oxygen atoms, which is consistent with the findings mentioned in these studies.

Titanium is widely distributed in the lunar maria and is one of the main components of lunar mare basalts. The role of Ti in the glass network structure has always been a significant focus for researchers. We have found that when the content of TiO_2_ is relatively low (0.5–3.5 mol%), there is basically no Q^0^ detected in the Raman spectrum’s *S_H_* region of basalt glass. However, when the TiO_2_ content reaches 6.5 mol%, there is a significant increase in Q^0^, while Q^4^ nearly disappears and *S_L_* decreases. Yang et al. [55] observed that with high TiO_2_ contents (21.9–27.9 wt%) in blast furnace slag glass, the monomers of [TiO_4_] tetrahedra increase as the TiO_2_ content increases, as determined through the analysis of high-temperature viscosity behavior and Raman spectroscopy. Guignard et al. [56] found that in the Mg–Al–Si–Ti system with a low TiO_2_ content (less than 6.0 mol%), titanium ions primarily form [TiO_4_] tetrahedra, and nuclear magnetic resonance confirmed that increasing the TiO_2_ content in this glass system leads to the substitution of Ti ions for T ions, forming partial T–O–Ti covalent bonds in the [TO_4_] tetrahedra. Osipov et al. [44] mentioned that in the Na–Ti–Si ternary system glass, with a large addition of TiO_2_ (5–30 mol%), a new peak was formed at around 884 cm^−1^ in the Raman spectrum. As the TiO_2_ content increased, the high-wavenumber envelope peak shifted toward lower wavenumbers. Based on the above literature and combined with our test analysis, a small amount of Ti can act as a network former and participate in the construction of the basalt glass network. However, as the Ti content increases to 6.5 mol%, some of the Ti forms separate [TiO_4_] tetrahedra, leading to a reduction in the glass network’s degree of polymerization. Additionally, the joint effect of Ti and Fe cannot be excluded.

Si and Al, as the primary formers in glass networks, play a crucial role in network construction [57]. The relationship between these elements in forming the glass network is integral, as evidenced by a significant body of Raman spectroscopy research. This research indicates that as the Si and Al content increases, the degree of polymerization within the glass network improves. This enhancement in polymerization can be observed in the corresponding peaks within the Raman spectra. Modifier cations, generally denoted as M^+^, are considered critical balancing cations within the structure of glass networks [58]. When there is a deficiency of M^+^ cations, [TO_4_] tetrahedra may force some excess T atoms (where T typically represents Si or Al) to adopt higher coordination numbers, forming [TO_5_] or [TO_6_] structures [59,60]. This shift is often due to the lack of sufficient balancing cations to maintain the preferred tetrahedral coordination. Furthermore, in conditions where the M^+^ cations are insufficient, it becomes easier for Fe^3+^ ions to be reduced to Fe^2+^ ions, which then act as network modifiers. This reduction and subsequent modification of the network can be evidenced by the IRI values, as observed in specific samples such as CE5. This process highlights the intricate balance and interplay between different components within the glass network, significantly influencing the network’s structure and properties.

### 3.3. Mechanical Properties

**Figure 5:**
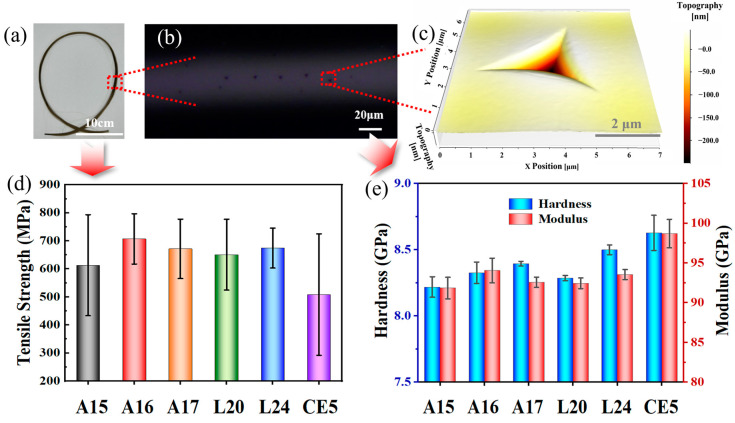
Test methods and results regarding the mechanical properties of basalt samples: (**a**) sample of simulated lunar sea basalt fiber; (**b**) nanoindentation test point on fiber; (**c**) scan of the indentation; (**d**) tensile strength test results; and (**e**) hardness and modulus test results.

Simulated lunar basalt was drawn into fibers with a diameter of 50 ± 5 μm using winding and speed adjustment, and their tensile strength was tested, as shown in Figure 5d. Upon analyzing the results, the following was found. Firstly, the lower ΔT values obtained through DSC testing might increase the likelihood of crystallization, leading to larger macroscopic defects in the fibers, resulting in lower tensile strength and greater variability. Secondly, the proportion of O_BO_ in each sample would affect the network polymerization degree, thus indirectly reflecting the macroscopic mechanical strength of the fibers. At the microscopic level, nanoindentation was employed to test the hardness and modulus, as illustrated in Figure 5a–c, which depict the nanoindentation test process of the simulated lunar basalt fibers. The testing points were distributed along the surface of the fiber axis, with 5 to 10 points tested on each fiber. The hardness and elastic modulus of each fiber were measured as shown in Figure 5e. CE5 exhibited significantly higher hardness and modulus compared to other samples, possibly due to the smaller bending amplitude of the Fe–O bonds. This also explains the weaker sensitivity of Raman spectroscopy to Fe–O bond identification mentioned earlier, as the abundant distribution of Fe–O bonds leads to smaller strain for the same force in the glass network structure.

## 4. Conclusions

Lunar basalt glass is a complex glass system where compositional fluctuations significantly influence the future preparation of fibers. Raman spectroscopy, in conjunction with other testing techniques, effectively analyzes and characterizes the glass network structure and its performance implications. This study employed the “peak area normalization” method of Raman spectroscopy. The spectral baseline calibration was uniformly set, and the displacement range of each fitted peak was converged and optimized to minimize the interference from subjective factors, facilitating lateral comparisons among samples within the same test batch.

This study shows that Fe^3+^ can substitute for Al or Si to construct T–O–T bridging oxygen bonds, which is notably reflected in the low wavenumber region (595 cm^−1^). Additionally, an increase in the iron content not only reduces the degree of polymerization in the glass network structure but also increases the potential for crystallization, consequently diminishing the macroscopic mechanical strength of the fibers, in agreement with the tensile test results. Surprisingly, Fe^2+^ serves as a beneficial network modifier when substituting for Na, Ca, and other cations to form non-bridging oxygen bonds, positively impacting properties such as the nanohardness and modulus.

Furthermore, we discovered that the [TiO_4_] tetrahedra formed by Ti participate in the construction of the basalt glass network. However, as the Ti content increases, as seen in samples like CE5, it tends to form Ti–O_NBO_ (non-bridging oxygen) bonds more frequently, reducing the degree of polymerization in the glass network. This reduction may be influenced by the combined effects of Fe and Ti. This also provides a reference for our future studies on the impact of Ti on the structure and properties of basalt glass networks.

## Figures and Tables

**Table 1:** Oxide components of each simulated lunar sea basalt/mol%.

Sample	SiO_2_	Al_2_O_3_	Fe_2_O_3_	TiO_2_	Na_2_O	K_2_O	MgO	CaO
A15	51.4	9.4	5.9	1.2	0.4	0.1	18.9	12.7
A16	50.4	18.0	2.1	0.5	0.5	0.1	9.6	18.8
A17	48.2	11.2	5.1	3.5	0.4	0.1	17.4	14.1
L20	48.7	14.2	2.8	0.5	0.5	0.1	15.8	17.4
L24	50.3	8.4	8.5	1.1	0.3	0.1	16.2	15.1
CE5	51.7	6.1	10.9	6.5	0.4	0.1	8.9	15.4

**Table 2:** Characteristic temperature of DSC.

Sample	T_g_/°C	T_c_/°C	ΔT/°C
A15	669.2	857.1	187.9
A16	729.6	909.5	179.9
A17	668.9	869.4	200.5
L20	703.4	933.0	229.6
L24	644.1	820.2	176.1
CE5	634.8	785.6	150.8

**Table 3:** Area proportion of each fitted peak.

Sample	*S_L_*/Σ*S/*%	*S_H_*/Σ*S/*%	Percentage of Each Q*^n^* in Σ*S*/%				
			Q^0^	Q^1^	Q^2^	Q^3^	Q^4^
A15	32.6	67.4	<0.1	10	26.1	25.3	6
A16	37.7	62.3	<0.1	7.6	26.1	23.9	4.7
A17	40.9	59.1	<0.1	8.9	24.4	20.4	5.5
L20	40.2	59.8	<0.1	8.4	24.1	20.3	7
L24	43.7	56.3	<0.1	10.3	22.8	17.3	5.8
CE5	24.1	75.9	24.7	24.7	19.5	6.9	<0.1

**Table 4:** IRI values of basalt fibers and the content of Fe*_x_*O*_y_*.

Samples	A15	A16	A17	L20	L24	CE5
IRI	0.24	0.21	0.25	0.19	0.21	0.27
FeO	2.8	0.9	2.6	1.1	3.6	5.9
Fe_2_O_3_	4.5	1.7	3.8	2.3	6.7	8.0

## Data Availability

The data presented in this study are available on request from the corresponding author.
